# Binnacle: Using Scaffolds to Improve the Contiguity and Quality of Metagenomic Bins

**DOI:** 10.3389/fmicb.2021.638561

**Published:** 2021-02-24

**Authors:** Harihara Subrahmaniam Muralidharan, Nidhi Shah, Jacquelyn S. Meisel, Mihai Pop

**Affiliations:** Pop Lab, Department of Computer Science, Center for Bioinformatics and Computational Biology, UMIACS, University of Maryland, College Park, MD, United States

**Keywords:** metagenomics, binning approach, metagenome assembly, strain variation, genome scaffolding

## Abstract

High-throughput sequencing has revolutionized the field of microbiology, however, reconstructing complete genomes of organisms from whole metagenomic shotgun sequencing data remains a challenge. Recovered genomes are often highly fragmented, due to uneven abundances of organisms, repeats within and across genomes, sequencing errors, and strain-level variation. To address the fragmented nature of metagenomic assemblies, scientists rely on a process called binning, which clusters together contigs inferred to originate from the same organism. Existing binning algorithms use oligonucleotide frequencies and contig abundance (coverage) within and across samples to group together contigs from the same organism. However, these algorithms often miss short contigs and contigs from regions with unusual coverage or DNA composition characteristics, such as mobile elements. Here, we propose that information from assembly graphs can assist current strategies for metagenomic binning. We use MetaCarvel, a metagenomic scaffolding tool, to construct assembly graphs where contigs are nodes and edges are inferred based on paired-end reads. We developed a tool, Binnacle, that extracts information from the assembly graphs and clusters scaffolds into comprehensive bins. Binnacle also provides wrapper scripts to integrate with existing binning methods. The Binnacle pipeline can be found on GitHub (https://github.com/marbl/binnacle). We show that binning graph-based scaffolds, rather than contigs, improves the contiguity and quality of the resulting bins, and captures a broader set of the genes of the organisms being reconstructed.

## Introduction

Advances in high-throughput sequencing strategies have spurred microbiome research and revealed important insights into the microbial communities that inhabit human, animal, and environmental habitats (The Human Microbiome Project Consortium, 2012; Oh et al., 2014; Zeevi et al., 2019). In particular, whole metagenomic shotgun sequencing, which allows for a comprehensive analysis of microbial DNA from a sample, has been instrumental in expanding our understanding of the functional potential and genetic composition of different microorganisms that have not been previously cultured. An important step in characterizing organisms that have not been isolated is the reconstruction of their complete genome sequence (Uritskiy and DiRuggiero, 2019; Mu et al., 2020). This process involves assembling short metagenomic reads into longer contiguous sequences (contigs) based on sequence overlap. Paired-end read information can then be used to link together and orient assembled contigs into scaffolds (Gao et al., 2011; Koren et al., 2011; Nurk et al., 2017; Ghurye et al., 2019). However, constructing the genomes of organisms from a mixture (metagenomic assembly) is computationally challenging. The uneven abundance of organisms, repetitive sequences within and across genomes, sequencing errors, and strain-level variations within a single sample often contribute to incomplete and fragmented assemblies.

In order to improve upon the fragmented assemblies constructed by metagenomic assembly tools, researchers utilize a strategy called binning, which involves clustering together genomic fragments that likely originate from an individual organism. Several strategies have been proposed for metagenome binning. Classification-based approaches rely on assigning taxonomic labels to genomic contigs, then grouping together those contigs that share a taxonomic label (Nguyen et al., 2014; Menzel et al., 2016; Von Meijenfeldt et al., 2019; Wood et al., 2019). Because many of the microbes found in microbial communities have yet to be characterized, classification-based approaches are limited to organisms (and genomic segments within) that are sufficiently related to known sequences. Clustering-based approaches focus instead on genomic features, such as GC content, oligonucleotide frequencies and contig abundance (coverage), to cluster together contigs that share similar properties (Tyson et al., 2004; Albertsen et al., 2013). While such approaches are effective even when an organism shares no similarity to any known sequences, they are stymied by genomic regions that have unusual DNA composition or that appear at higher depth of coverage than other segments of the organism of interest – situations that frequently occur in plasmids, mobile genetic elements, and highly conserved genomic segments (such as the 16S rRNA operon) (Arredondo-Alonso et al., 2017).

Clustering/binning has also been applied to genes rather than contigs (Bjørn Nielsen et al., 2014). The resulting clusters were termed co-abundance gene groups (CAGs). CAGs that contained a large number of genes, roughly equivalent to the expected number of genes in a bacterial genome were referred to as metagenome species (MGS). More recently, in metagenome binning, when a cluster of contigs represents a complete, or close to complete, genome, it is referred to as a “metagenome-assembled genome” (MAG). While it is possible to recover MAGs from automated metagenome binning algorithms, many of the clusters obtained are incomplete or contaminated, and manual “finishing steps” are required to recover MAGs. In this paper, because we work with clusters obtained directly from binning algorithms, we refer to them as metagenomic bins rather than MAGs unless, referring to high quality bins.

While scaffolding and binning are both approaches for grouping together contigs that belong to an individual organism, they are often applied independently of each other, with some exceptions. MaxBin (Wu et al., 2014), for example, uses genomic scaffolds as a substrate for binning, however, they appear to be handled as if they were linear contigs. A newer version of this tool, MaxBin 2.0 (Wu et al., 2016), focuses solely on contigs. COCACOLA (Lu et al., 2017) incorporates paired-end information as another source of linkage information during the binning process, and does not explicitly construct or leverage scaffold information. GraphBin2 (Mallawaarachchi et al., 2020) independently bins contigs then refines the bins in the context of an assembly graph, by correcting bin assignments and propagating labels to unbinned nodes in the graph.

Here, we demonstrate the effectiveness of explicitly accounting for scaffold information in binning. We describe novel algorithms for estimating scaffold-level depth of coverage information that are effective even for non-linear (graph) scaffolds, and show that variation-aware scaffolders, which detect and explicitly model ambiguity in the assembly graph, help further improve the completeness and quality of the resulting metagenomic bins. We present a new software tool, Binnacle that accurately computes coverage of graph scaffolds and seamlessly integrates with leading binning methods. We show that using graph scaffolds for binning improves the contiguity and quality of metagenomic bins and captures a broader set of the accessory elements of the reconstructed genomes. Binnacle is implemented in Python 3 and released open source on GitHub at https://github.com/marbl/binnacle.

## Materials and Methods

**FIGURE 1:**
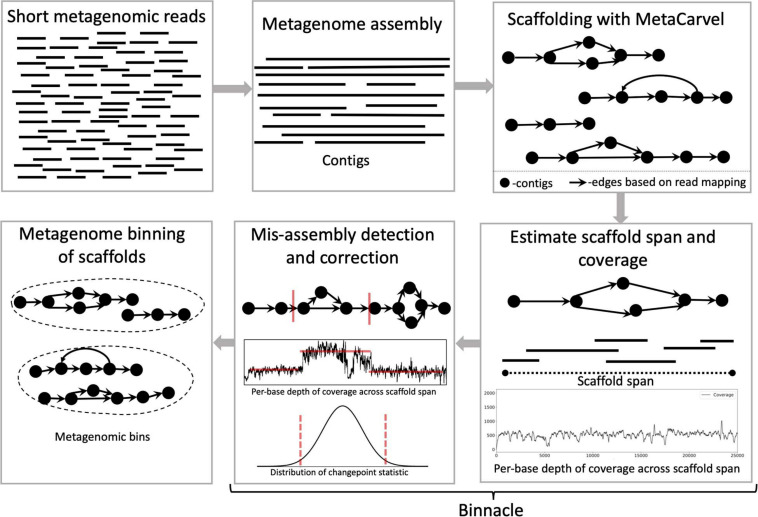
Schematic diagram of the Binnacle pipeline. Short reads are assembled into contigs with a metagenome assembly tool. These contigs are oriented and ordered to generate graph scaffolds. For each scaffold, based on the length, orientation, and gap estimates, each contig in a scaffold is assigned global start and end coordinates; and the span of the scaffold is computed. Scaffold coverage is the per-base depth of coverage across the scaffold span. In the mis-assembly detection and correction routine, scaffolds are broken up if there are discontinuities in coverage signals. The final set of scaffolds and corresponding coverage information are used as input to binning methods to generate metagenomic bins.

Binnacle operates as an add-on to existing binning tools. It relies on MetaCarvel (Ghurye et al., 2019) to construct genomic scaffolds, then uses a new algorithm for estimating the depth of coverage/abundance of scaffolds from read-mapping data, taking into account genomic variation as well as potential mis-assemblies and other artifacts. The resulting abundance information across one or more samples is then provided to a binning algorithm in order to generate scaffold-level bins (Figure 1). Each step in this pipeline is described in more detail below.

### Metagenome Assembly

Like other binning approaches, Binnacle relies on the output of a metagenomic assembler. Any metagenomic assembler can be used to assemble the data, with the caveat that assembly errors can have a significant negative impact on binning. The results presented in this paper were generated by assembling each sample separately (i.e., avoiding a possibly expensive co-assembly step), and details about the tools and parameters used are presented below.

### Scaffolding With MetaCarvel

Sequencing reads are mapped back to the assembled contigs, and the paired-end read information is used to scaffold the contigs using MetaCarvel (Ghurye et al., 2019). This process results in a scaffold graph, where nodes are contigs and edges represent contig adjacencies inferred from paired-end read information. The scaffold graphs constructed by MetaCarvel are non-linear and preserve complex graph patterns, such as bubbles, which manifest when contigs diverge into one or more paths before converging again. Such patterns typically correspond to sequence variants between closely related organisms within a community, such as insertion/deletion (indel) events. Binnacle specifically works with the MetaCarvel scaffolder because of its unique ability to preserve variation in scaffolds.

### Estimating Scaffold Span and Coverage

One of the key features used by binning algorithms is information about the abundance/depth of coverage of genomic contigs, either within a single sample, or across multiple samples. To our knowledge, coverage estimation of scaffolds within metagenomic data sets has not been critically explored. Most current approaches rely on raw read counts averaged across the contigs or scaffolds being binned, similar to the “reads per kilo-basepair per million” (RPKM) measure used in RNA-seq analysis. A number of artifacts impact coverage estimation from scaffolds using such an approach, including potential overlaps between contigs (particularly relevant within regions of genomic variation), and assembly or scaffolding errors.

**FIGURE 2:**
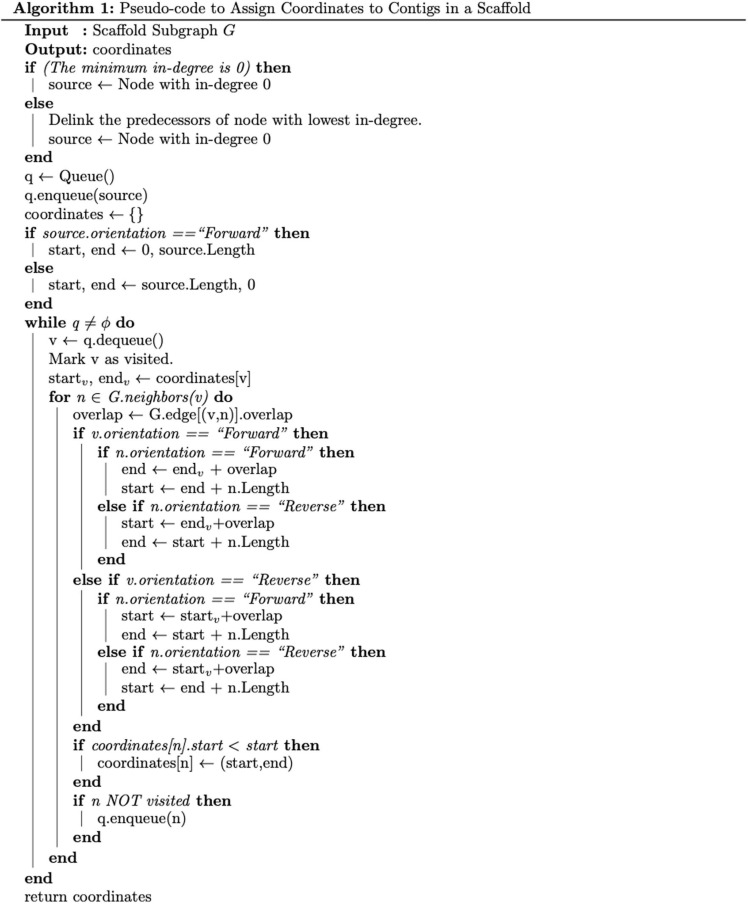
Assigns start and end coordinates to contigs in a scaffold. The lowest start coordinate and the highest end coordinate determine the scaffold span.

In non-linear “graph” scaffolds, such as those generated by MetaCarvel, the genomic extent covered by the scaffold cannot be directly inferred from the size of the contigs that are scaffolded together. To estimate the scaffold span – total effective length of the scaffold, i.e., the distance from the starting contig to the maximal rightmost coordinate of contigs contained in the scaffold – we rely on the following algorithm. For every graph scaffold, we identify a node with in-degree 0 which is assigned coordinate 0. If a scaffold contains no nodes with in-degree 0, we break the cycle using an approximation of the minimum feedback arc set problem. This problem is known to be NP-complete (Berger and Shor, 1990; Even et al., 1998) and hence we use an approximate solution: delinking the incoming edges of a vertex with the lowest in-degree. Coordinates for the other contigs in the scaffold are assigned in a breadth-first manner taking into account the length of the contig, the length of overlap between contigs, and the relative orientation of the contigs (Figure 2). If there are multiple possible coordinate assignments for a contig (vertex), we retain the one with the largest possible value. We use this heuristic because choosing any other strategy to break ties might lead to an artificial increase in depth of coverage and negatively impact coverage computation. The span of the scaffold is then assigned to the distance between the right-most and left-most ends of the scaffolded contigs, based on the inferred contig coordinates.

Once the coordinates are available, we map reads to the contigs using Bowtie 2 (version 2.3.0) (Langmead and Salzberg, 2012) and estimate per-base contig coverage using the genomecov program in the bedtools (version 2.26.0) suite with the options -bga and -split. The per-base coverage of the scaffold is computed by adding up the coverage information of the contigs that overlap at each position in the scaffold span.

### Detection and Correction of Mis-Assemblies

When building graph scaffolds, MetaCarvel uses mapping of paired-end reads to contigs to infer adjacency information, however, this approach can sometimes falsely link together contigs. To detect such events, we rely on discontinuities in the depth of coverage signal as follows.

Ignoring sequencing biases, we expect each genomic position within a scaffold span to be covered equally well (uniformly). Hence, we assume that the per-base coverage of each organism (scaffold) follows a Poisson distribution and can be approximated by a Gaussian distribution with a mean, μ and a variance, σ^2^. To break up scaffolds containing contigs possibly originating from multiple species, we rely on a change point detection algorithm (Adams and MacKay, 2007; Aminikhanghahi and Cook, 2017) that operates on the per-base coverage signals.

To identify change points, we slide a window *w* of size |*w*| along the coverage signal, computing the empirical means and variances. The user can select any value of *w*, but by default, we set |*w|* = 1500 bp. For scaffolds shorter than 3000 bp, we recursively set |*w|* = |*w|* /5 until the scaffold length is at least 2*w*. For each position *i* along the scaffold span, we note the mean μ_*i*−1_ and variance σ2i-1 of the window *w*_*i–1*_ defining the coverages from the coordinates *i*−|*w*| to *i* and the mean μ_*i*_ and variance σ2i of the window *w*_*i*_ defining the coverage along the positions from *i*to *i* + |*w*|. We identify the windows *w*_*i–1*_ and *w*_*i*_ with respect to the position *i* as predecessor and successor windows, respectively. Given the coverage distribution for the two windows, we compare these distributions using the two-sample Z-statistic given by,

Z=μi-1-μiσ2i-1+σ2i

The empirical distribution of the Z-statistic such derived forms a Gaussian distribution, and we select the points within the tails of the Z-statistic distribution as candidates for change points (by default, we set α = 1 percentile). To reduce the potential for false-positives, we next check if the change points coincide with the start or end of a contig within the scaffold, which suggest that the identified contig is incorrectly linked into the scaffold. Therefore, we delink the contig from its predecessors if the change point coincides with its start and delink from its successor if the change point coincides with its end (β = read length). We also note that there are a few change points identified by our algorithm that do not coincide with the start or end of a contig. These could be due to either statistical artifacts or errors introduced by the assembler, but we do not currently address these in Binnacle.

**FIGURE 3:**
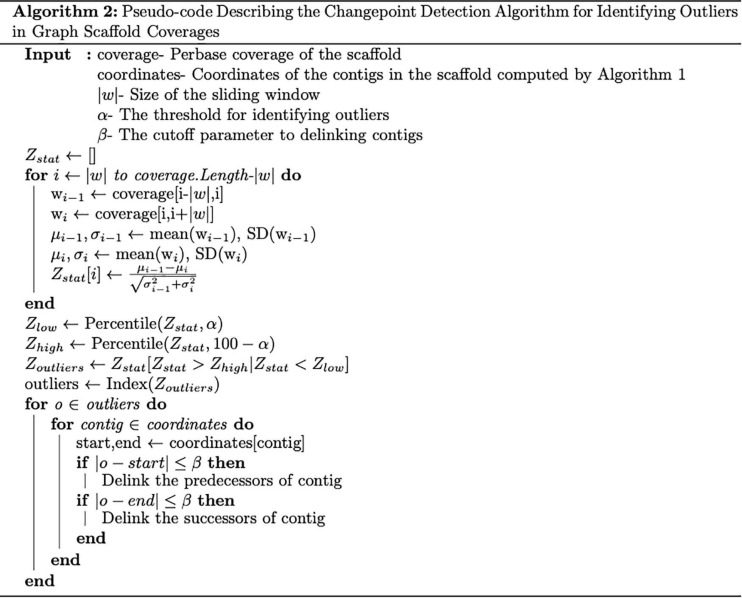
Pseudocode describing the change-point detection algorithm. The algorithm takes in two parameters α and β denoting the threshold for identifying outliers and the cutoff parameter to delink contigs, respectively.

**FIGURE 4:**
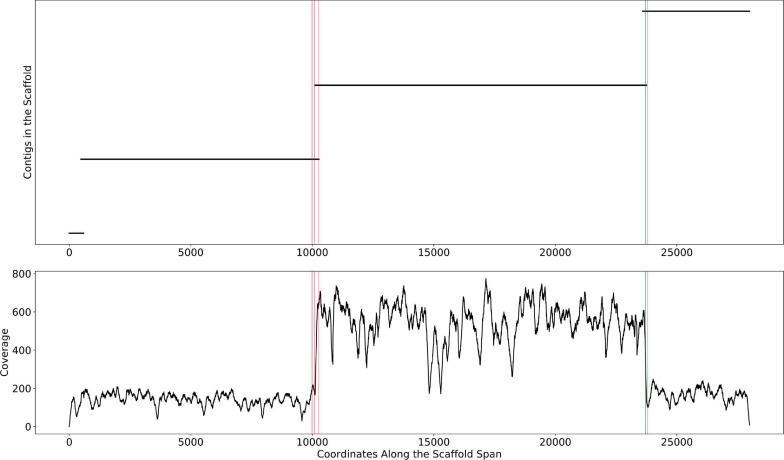
The mis-assembly detection algorithm in Binnacle. This is a scaffold from HMP sample SRS012902. The plot on the top shows the position of contigs along the scaffold span. The plot at the bottom shows the per-base depth of coverage across the scaffold span. The locations detected by the change point detection algorithm are highlighted by vertical red lines.

This change point detection algorithm can work with both contig and scaffold coverages. We note that 40% of the time, a change point coincides with the beginning or end of a contig. When this happens, we delink the contig in the scaffold (i.e., remove the connections between the contig and its neighbors, resulting in multiple scaffolds). The remaining 60% of change points either occur too close to a previously delinked contig or occur in the middle of contigs, revealing potential assembly errors. The handling of such situations requires further research that goes beyond the scope of this manuscript. The algorithm is described in detail in Figure 3. An example of the algorithm applied to a scaffold in the HMP dataset is shown in Figure 4. In the HMP dataset, an average of 4% of all the scaffolds were broken by change point detection.

After correcting potential scaffolding errors, Binnacle generates files reporting the per-base coverage for all scaffolds, describing the global coordinate information and describing the mean and standard deviation in coverage for all the scaffolds. In addition, we also provide a FASTA file of the final set of scaffolds after the mis-assembly detection routine. The abundance file and the scaffolds file provided by Binnacle can be readily used by existing binning algorithms. We currently provide interfaces to MetaBAT2 (Kang et al., 2019), MaxBin 2.0 (Wu et al., 2016), and CONCOCT (Alneberg et al., 2014).

### Estimating Scaffold Coverage Across Multiple Samples

The procedure described above is used when estimating scaffold coverage within the sample from which the scaffold is derived. If multiple samples are available, binning algorithms can leverage coverage information from all the samples to identify contigs/scaffolds that co-vary in abundance. When using multiple samples, the reads from each sample are mapped to the contigs/scaffolds of all of the samples and the mean abundance of each contig/scaffold is reported on a per sample basis. This approach produced fewer high contamination bins than binning without combining coverage information from multiple samples (Supplementary Figure 1). Identifying and comparing contigs across samples is challenging. Determining how to best use abundances estimated from multiple samples remains an active area of research.

### Analysis of Metagenomic Datasets

To benchmark Binnacle, we first relied on a known-composition mock dataset described in Kyrgyzov et al. (2020a), which is referred to as “simulated data” in the remainder of this paper. The corresponding data were obtained from the GigaDB database (Kyrgyzov et al., 2020b). We also evaluated our method on three real metagenomic datasets: (1) a time series of 18 fecal samples from a single premature infant (infant 31) from Sharon et al. (2013) referred to as the “infant gut data” in the remainder of this paper, (2) 20 complex stool samples from the Human Microbiome Project (The Human Microbiome Project Consortium, 2012) referred to as the “HMP gut data,” and (3) a time series of 12 samples from subject HV12 in a skin microbiome study (Oh et al., 2016) referred to as the “skin longitudinal data.” All three datasets are complex, human-associated microbiomes. The infant gut data was selected because there is good understanding of the underlying community structure and the study assembled and published several reference genomes^1^ of organisms identified within these samples. For the three real metagenomic datasets, we downloaded reads from the NCBI read archive. Supplementary Table 1 provides a list of accessions from each dataset.

For the HMP gut dataset, we used IDBA-UD assemblies provided by the HMP consortium. For all other datasets, we assembled the reads into contigs using MEGAHIT (version 1.1.2) (Li et al., 2016). For all datasets, we generated scaffolds using MetaCarvel (Ghurye et al., 2019). Both MetaCarvel and MEGAHIT were run with default parameters. MetaCarvel outputs both variation-aware graph scaffolds and optimized linear sequences as linear scaffolds. Through Binnacle, a mis-assembly detection and correction routine was used to break up any mis-joined scaffolds, and then scaffold coverages were estimated. We refer to scaffolds obtained through the Binnacle step as “graph scaffolds” and linear sequences from MetaCarvel as “linear scaffolds.”

To assess the quality of binning, in the simulated data set we relied upon the known genome sequences from which this dataset was constructed. Similarly, the publication describing the infant gut dataset identified a set of 33 reference genomes that were present in these samples, which we use as a reference for validation. In both datasets, we aligned the binned contigs to the reference genomes using minimap2 (version 2.1) (Li, 2018). Each bin was assigned to the genome to which the majority of base pairs aligned. We compute completeness as the percentage of the assigned genome represented in the bin, and contamination as the percentage of base pairs in the bin that did not align to the assigned genome. For the HMP gut data and the skin longitudinal data, where reference genomes were not available, we used CheckM (version 1.0.11) to compute the completeness and contamination of the bins.

In the simulated dataset, we tested three binning methods – MaxBin 2.0 (version 2.2.5) (Wu et al., 2016), COCACOLA (Lu et al., 2017), and MetaBAT2 (version 2.12.1) focusing on three features: contigs, linear scaffolds, and graph scaffolds. All methods employ a different threshold on the length of contigs used for binning. To make comparisons across binning methods fair, we ran MaxBin 2.0, COCACOLA, and MetaBAT2 with the same contig threshold (>2500 bp). COCACOLA can use paired-end information to assist binning. To assess the effectiveness of this feature we ran COCACOLA in paired-end mode on the assembled contigs. When applied to graph scaffolds and linear scaffolds, we disabled COCACOLA’s paired-end processing.

MetaBAT2 generated bins with lower contamination than both MaxBin 2.0 and COCACOLA (discussed later in results). Hence, for the three real metagenomic datasets, we only show results obtained with MetaBAT2 (Kang et al., 2019) (default parameters). MetaBAT2 uses the abundances and sequence composition information to bin genomic sequences. We estimated the coordinates, span, and abundance of scaffolds using Binnacle for each sample with its own set of reads. We then estimated abundances for each scaffold along the scaffold span using the reads of all other samples in the dataset as additional features. Similarly, while binning with contigs and binning with linear scaffolds, we computed mean and variance of coverages from all samples.

To examine bins in the skin longitudinal dataset, we focused on bins that belonged to the *Cutibacterium* (*Propionibacterium*) genus, as identified by CheckM (Parks et al., 2015). We extracted the contigs within each bin and aligned them to the *Cutibacterium acnes KPA171202* reference genome (GCA_000008345.1) using MetaQUAST (Mikheenko et al., 2016). Contigs within linear and graph scaffolds were used (instead of the scaffold sequences) to prevent misalignment of structural variant features. For pangenome analyses, a total of 27 complete *C. acnes* reference genomes were downloaded from NCBI (See Supplementary Table 2 for accession numbers). Genes were predicted from these references using Prokka (Seemann, 2014) and the pangenome was calculated using Roary (Page et al., 2015). Genes found in all 27 references were considered “core” genes and those found in at least 2 samples were considered “accessory.” Genes were predicted in the MAGs using Prodigal (Hyatt et al., 2010) with the “-p meta” option and were aligned using BLAST (Altschul et al., 1990) against the pangenome reference sequences (E-value 1e-3, percent identity 75). BLAST hits with a query and subject coverage of at least 50% were retained and annotated as either “core” or “accessory” genes. Genes with multiple hits were assigned to the hit with the greatest alignment length and percent identity. Genes identified in the metagenomic assemblies but not found in the reference genomes were flagged as “putative-accessory” genes. CRISPR/Cas elements were detected within the bins using CRISPRCasFinder on the web (Couvin et al., 2018). Contigs in MET0773 were annotated using Prokka v 1.12 (Seemann, 2014) and visualized with the R package genoPlotR (Guy et al., 2010).

## Results

To determine whether graph scaffolds can improve binning quality, we analyzed one simulated dataset and three sets of real metagenomic samples: infant gut samples, HMP gut samples, and skin longitudinal samples, described further in Methods. For samples from each of these datasets, we assembled and binned contigs and scaffolds with Binnacle and MetaBAT2.

### Impact of Accurate Estimation of Scaffold Coverage/Abundance

**FIGURE 5:**
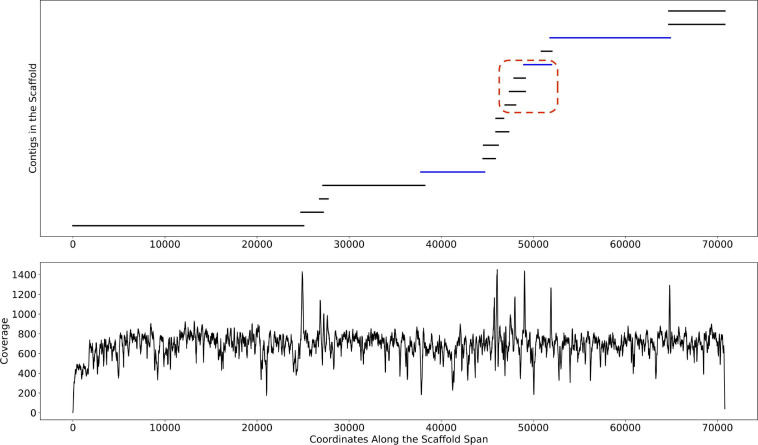
An example scaffold with coverage estimated with Binnacle. The plot at the top shows the position of contigs along the scaffold span. Contigs within the red dotted box are part of a bubble (signature of strain variation) detected by MetaCarvel. Only three contigs (highlighted in blue color) were binned by MetaBAT2 when contigs rather than scaffolds were provided as input. The plot at the bottom shows the cumulative per-base depth of coverage across the scaffold span as estimated by Binnacle.

Depth of coverage information is one of the key features used by binning algorithms. Correctly estimating this information is difficult, particularly in metagenomic datasets where genomic variants and highly conserved regions confound the signal. As described in Methods, Binnacle leverages information about the relative placement of contigs inside of a scaffold to better estimate abundance. As seen in Figure 5, the coverage signal estimated by Binnacle across the scaffold span of a single scaffold from the HMP stool sample SRS023829 is fairly uniform. This signal takes into account the overlap between multiple contigs, aggregating the coverage information within the overlapping region. The contigs from this scaffold can be assigned to organisms from the *Bacteroides* genus through a BLAST (Altschul et al., 1990) search against the nt database. When using contigs alone for binning, only three of these contigs were binned (highlighted in blue color in Figure 5). Some of the unbinned contigs may have been excluded due to their size as, by default, MetaBAT2 only bins contigs greater than 2,500 base pairs. However, there were also several long contigs that remained unbinned despite having strong paired-end read connections to the rest of the contigs.

### Binnacle Improves Contiguity, Completeness, and Contamination of Bins

To assess the effectiveness of different types of information in binning, we provided binning algorithms with three sources of data: (i) contigs (the most common usage); (ii) linear scaffolds; and (iii) graph scaffolds that preserve the ambiguity introduced in the assembly graph by genomic variation. The comparison between linear scaffolds and graph scaffolds allows us to determine whether any improvement in binning effectiveness is due to the longer sequences provided to binning algorithms, or if there is a real benefit in accounting for the structure of the graph in regions of genomic variation.

**FIGURE 6:**
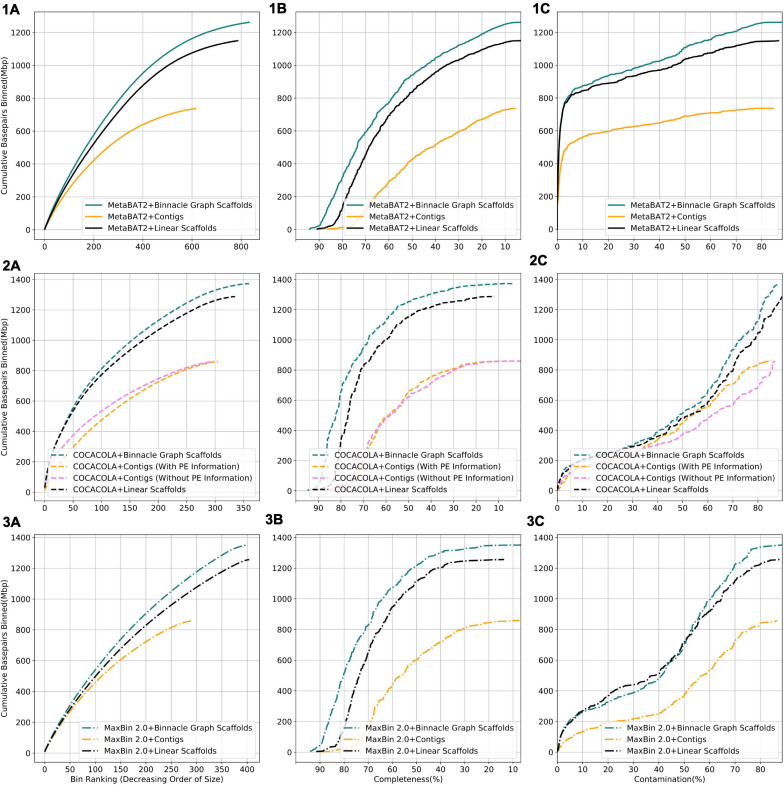
Binning with graph scaffolds improves contiguity, completeness, and contamination in genome bins from the simulated dataset. Comparing bins generated by MetaBAT2 (solid lines) (1), COCACOLA (dotted lines) (2), and MaxBin 2.0 (dashed-dotted lines) (3) using contigs (yellow), linear scaffolds (black), and graph scaffolds (blue) for the simulated dataset. COCACOLA contigs were binned both with and without paired end information. **(A)** Cumulative base pairs binned with contigs, linear scaffolds, and graph scaffolds. Bins are ordered in decreasing order of their size. The upper curve corresponds to higher contiguity for the same number of bins. **(B)** Completeness is defined as the percentage of the assigned genome represented in the bin. Bins are ordered in decreasing order of their completeness value. The upper curve indicates that more base pairs are binned by graph scaffolds at the same or higher level of completeness. **(C)** Contamination of a bin is defined as the percentage of base pairs that did not align to the assigned genome. Bins are ordered in the increasing order of their contamination value. The higher curve indicates that more base pairs are binned by graph scaffolds at the same or lower level of contamination.

We compared results from three binning methods, MaxBin 2.0, COCACOLA, and MetaBAT2 each supplied with contigs, linear scaffolds, or graph scaffolds. For all three methods, bins generated with graph scaffolds comprised more base pairs, and had higher completeness and lower contamination than bins generated with contigs or with linear scaffolds (Figure 6). The simulated dataset contained 100 genomes. We aligned contigs from each bin to the known reference genomes and taxonomically annotated bins with the genome for which the majority of base pairs aligned. To ensure only one bin per reference genome, we only considered bins that were at least 50% complete. Graph scaffolds, linear scaffolds, and contigs recovered 40, 38, and 21 putative genomes on average, respectively. In the case of COCACOLA, a tool that can leverage paired-end information natively, we observed that its handling of this information was less effective than that provided by scaffolding approaches such as MetaCarvel (the basis for the scaffolds used in Binnacle) (second row in Figure 6). Moreover, when using paired-end information, contiguity and completeness were comparable; only contamination of the bins was improved. Irrespective of the binning method employed, graph scaffolds improved the contiguity, completeness, and contamination of the resulting bins. However, we used MetaBAT2 as the binning method for the remaining analyses in this paper.

We assessed both the completeness and level of contamination of the resulting bins from all three real metagenomic datasets. For the infant gut dataset, we computed completeness and contamination of the bins based on a set of 33 reference genomes that were identified to be present in these samples (See section “Materials and Methods”). Similar to the performance on simulated data, bins generated with graph scaffolds contained more base pairs than bins generated with contigs and linear scaffolds (Supplementary Figure 2). Moreover, bins from graph scaffolds had higher completeness and lower contamination than bins generated with contigs and linear scaffolds.

**FIGURE 7:**
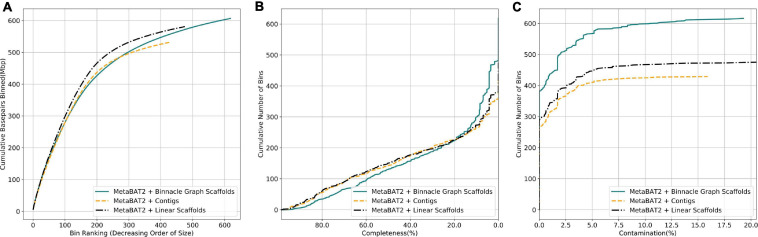
Graph scaffolds bin more contigs and reduce bin contamination in the HMP gut dataset. Comparing bins generated by MetaBAT2 using contigs, linear scaffolds, and graph scaffolds for the HMP gut dataset. The completeness and contamination of bins were evaluated with CheckM. **(A)** Cumulative base pairs binned with contigs, linear scaffolds, and graph scaffolds. Bins are ordered in decreasing order of their size. The upper curve corresponds to higher contiguity for the same number of bins. **(B)** Bins are ordered in decreasing order of their completeness value from CheckM evaluation. The upper curve indicates that more bins are at the same or higher level of completeness. **(C)** Bins are ordered in the increasing order of their contamination value from CheckM evaluation. The higher curve indicates that more bins are at the same or lower level of contamination.

We next analyzed complex metagenomic samples from the HMP gut study. We did not have prior information about the community structure and genomes present, so we used CheckM (Parks et al., 2015) to evaluate the bins. CheckM uses sets of highly prevalent single-copy genes to assess the overall quality of genomes or genome bins, including their completeness, contamination, and strain heterogeneity. Bins generated from linear scaffolds grouped more base pairs than bins generated with contigs (Figure 7A). They also had comparable completeness and generally lower contamination (Figures 7B,C). When using graph scaffolds that include potential strain variants, the contiguity of the resulting bins improved, and a majority of bins have low contamination level (Figure 7, solid blue line).

Samples in the HMP gut dataset contained an average of 70 million reads. Binnacle took an average of 7.75 min to run (min = 2.7, max = 96.75, SD = 31.75 min) and had a peak memory usage of less than 3GB on average (min = 1.6, max = 10, SD = 2.57 GB). The run time and memory requirements on the HMP gut samples are shown in Supplementary Table 4. We ran these samples on a Linux computing cluster specifying a memory limit of 36 GB using a single processor. Given that these jobs took less than 10 GB of memory to run, they should run efficiently on most modern computing hardware.

### Binnacle Recovers *Cutibacterium acnes* Bins From Sebaceous Skin Samples

To further evaluate Binnacle’s performance, we used it to bin the skin longitudinal dataset with multiple samples from two sebaceous, or oily, skin sites – the back of the head (occiput) and the external auditory canal of the ear – as well as two moist body sites – the toe web and plantar heel – all from the same healthy volunteer. Within these samples, there were similar improvements in bin contiguity, completeness, and level of contamination when binning graph scaffolds compared to when binning contigs and linear scaffolds (Supplementary Figure 3).

*Cutibacterium acnes*, formerly referred to as *Propionibacterium acnes*, is a known prominent bacterial community member at sebaceous skin sites because it utilizes the fatty acids in the sebum (the oily substance produced by sebaceous glands) for energy. Different strains of the commensal *C. acnes* have been associated with acne vulgaris (Fitz-Gibbon et al., 2013). Because of its prominence on the skin and its implications for skin health, we searched for this organism in the skin longitudinal dataset; we were able to recover bins belonging to the *Cutibacterium* genus from five of the six sebaceous samples (Table 1). These bins contained contigs belonging to *C. acnes*. We mapped the *Cutibacterium* bins to the reference genome for *C. acnes* and found that bins generated with graph scaffolds generally covered a greater proportion of the reference genome than bins generated with contigs and linear scaffolds. Furthermore, both linear and graph scaffolds were able to recover a *Cutibacterium* bin from sample MET0754 that was not identified when binning with contigs alone.

**TABLE 1:** *Cutibacterium* bins detected in the skin longitudinal samples.

**Body site**	**Timepoint**	**Sample**	**Method**	**# Contigs**	**# Contigs (>1,000 bp)**	**Total length**	**Total aligned length**	**Genome fraction (%)**	**# of bubble contigs**
External auditory canal (Ea)	1	MET0308	contig	367	367	2,290,385	2,136,612	80.191	12
			linear scaffold	591	493	2,475,611	2,390,694	87.702	43
			graph scaffold	669	520	2,606,365	2,475,094	88.404	52
	2	MET0749	contig	237	237	2,601,507	2,452,477	93.375	4
			linear scaffold	288	256	2,662,358	2,502,296	94.439	7
			graph scaffold	305	260	2,680,429	2,514,146	94.495	7
	3	MET0768	contig	136	136	2,548,346	2,444,275	94.219	2
			linear scaffold	120	108	2,506,265	2,447,111	94.6	3
			graph scaffold	160	137	2,370,487	2,262,214	86.668	4
Occiput (Oc)	2	MET0754	linear scaffold	1059	671	1,711,485	1,559,541	57.717	0
			graph scaffold	972	625	1,606,432	1,463,457	54.226	0
	3	MET0773	contig	365	365	1,850,617	1,782,219	67.091	5
			linear scaffold	742	546	2,342,529	2,183,639	77.716	41
			graph scaffold	966	677	2,777,243	2,460,422	81.625	73

*Bins identified as *Propionibacterium* by CheckM were found in five total samples from two different sebaceous (oily) body sites. Contigs within the bins were aligned to the *Cutibacterium acnes KPA171202* reference genome (GCA_000008345.1) and stats were generated using MetaQUAST (Mikheenko et al., 2016).*

**FIGURE 8:**
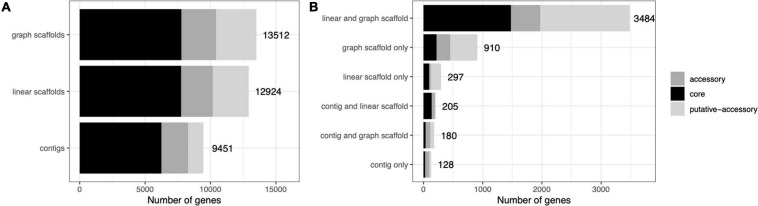
*Cutibacterium* bins generated by graph scaffolds capture more auxiliary genome elements. Genes predicted from *C. acnes* bins were mapped to genes from the *C. acnes* pangenome and characterized as core, accessory, or putative-accessory. The *x*-axis denotes the number of genes in all of the *C. acnes* bins and the *y*-axis denotes the method by which each gene was binned. The label denotes the total number of genes in each bar. In **(A)** all genes binned by each method are included in the bars, while in **(B)** they are separated by how they are shared across binning methods.

A common concern with binning algorithms is that they largely capture the core genome of organisms, omitting potentially relevant accessory genes. We classified *C. acnes* genes into core, accessory, and putative-accessory genes as described in Methods. As seen in Figure 8, bins constructed from graph scaffolds captured a larger fraction of accessory and putative-accessory genes, while bins constructed from contigs (the most commonly used approach) contained mostly core genes. Among the accessory and putative accessory genes identified in the metagenomic assemblies, 86.9% were binned within graph scaffold bins (10.5% were uniquely binned by graph scaffolds and no other methods).

### Binnacle Captures Structural Genomic Variation

By using scaffolds that include structural variants, we intended to capture genes and genomic elements that are typically missed by contig-based analyses. As shown in Table 1, many contigs identified within variant regions by MetaCarvel appeared only in bins constructed from these scaffolds, i.e., the information typically used by binning algorithms was not able to associate these contigs with the *C. acnes* genome.

**FIGURE 9:**
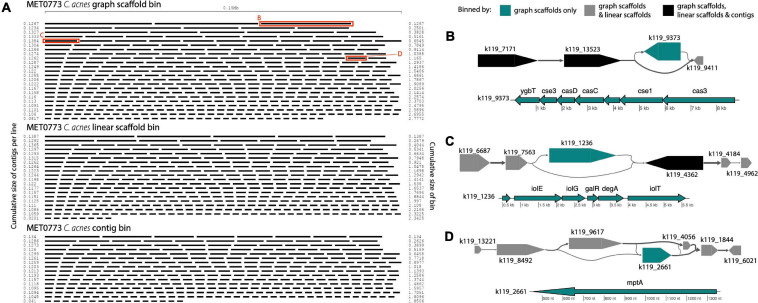
*Cutibacterium* bins in sample MET0773. **(A)** Ordered lengths of graph scaffolds (top), linear scaffolds (middle) and contigs (bottom) included in *C. acnes* bins, highlighting the greater fragmentation in the bin generated using contigs. Red boxes highlight graph scaffolds depicted in parts **(B**–**D)**. In **(B–D)**, the large arrows represent contigs in a single graph scaffold. Lines connecting contigs denote paired-end read support. Contigs are colored to indicate the methods that include them in the *C. acnes* bins. Scaffold plots were generated by MetagenomeScope (Fedarko et al., 2017) but updated and modified to improve visualization in Illustrator. Genes in contigs uniquely binned by graph scaffolds are depicted below the scaffold as thin arrows. Genes were predicted and annotated by Prokka (Seemann, 2014) and visualized with the R package genoPlotR (Guy et al., 2010).

In sample MET0773, all three scaffolding methods detected a *C. acnes* bin (Table 1), however, the *C. acnes* bin generated using graph scaffolds was more contiguous and had less fragmentation than the bin generated using contigs (Figure 9A). Furthermore, a total of 32 variant contigs (2 indels, 20 simple strain variants, and 10 complex strain variants) were uniquely identified in the *C. acnes* bin generated using graph scaffolds. One such variant contained elements of the subtype I-E CRISPR-Cas system (Figure 9B) that has previously been characterized in *C. acnes* (Brüggemann et al., 2012). Within this same sample, a contig that was not in a structural variant but was uniquely binned using graph scaffolds contained a CRISPR array with five spacers, one of which had close similarity to the *Cutibacterium phage PAVL21* genome (Supplementary Table 3). Another indel that was only binned by graph scaffolds contains genes involved in the degradation of myo-inositol into acetyl-CoA (Figure 9C). In *Corynebacterium glutamicum*, genes involved in this pathway allow the bacterium to use myo-inositol as a carbon and energy source (Krings et al., 2006). This indel also contains genes encoding two HTH-type transcriptional regulators (galR and degA). A contig uniquely binned by graph scaffolds in a complex strain variant contains a gene annotated as mptA (Figure 9D); in *Mycobacterium tuberculosis* and *C. glutamicum*, this gene is involved in the biosynthesis of cell-wall associated lipomannan that has several immunomodulatory properties (Mishra et al., 2007, 2011).

## Discussion

Binning (based on sequence composition and depth of coverage) and scaffolding (based on paired-end information) provide complementary approaches for grouping together contigs from metagenomic samples that likely originate from the same organism. At the outset of our study, we hypothesized that combining the two approaches would yield improvements in the contiguity and quality of the resulting bins. While others have used paired-end read or scaffold information to augment binning, we identified a major overlooked factor – the computation of depth of coverage at a scaffold level, computation that can be impacted by scaffolding errors and strain variation. To our knowledge this contribution is novel, and as we have shown, providing binning algorithms with depth of coverage information derived from linear and non-linear (graph) scaffolds improves the quality of the bins over what can be achieved by binning contigs alone.

We attribute the improvements we have demonstrated to three factors. The first is, as already mentioned, a more accurate estimation of scaffold depth of coverage, information used by the binning algorithm to determine which contigs or scaffolds should be grouped together. The second is simply the longer-range information available in scaffolds as opposed to individual contigs. A third factor is the use of variation-aware scaffolds which were referred to as “graph scaffolds” in the manuscript.

Binning algorithms rely on depth of coverage and sequence composition information, and accurately estimating this information requires long genomic segments. As a result, small contigs get excluded from binning either by design or because of incorrect estimates of coverage or sequence composition. The longer genomic context of scaffolds provides an opportunity for binning algorithms to more accurately estimate the information necessary for binning. Furthermore, certain genomic regions, such as mobile elements, usually have a different sequence composition from the rest of the genome (this is in fact one of the signals used to detect such regions) and may, therefore be missed. Paired-end information, however, can link together contigs irrespective of length and sequence composition, thereby capturing a larger fraction of the sequence from the assembly. These links are generally accurate; in the simulated dataset over 99% of the paired-end reads linked contigs belonging to the same species (Supplementary Figure 4).

Typically, metagenome assemblers and scaffolders attempt to construct a single linear sequence representing a segment from the chromosome of an organism in the sample. In many cases, however, such a linear representation ignores the presence in the sample of multiple variants of an organism, not unlike the presence of multiple isoforms of genes in eukaryotic transcriptomes. By explicitly modeling this variation, Binnacle is able to more accurately estimate the depth of coverage of scaffolds, thereby improving the efficacy of the binning process. When considering only a linear representation of a contig or scaffold, conserved genomic regions would appear to have higher depth of coverage than the variant regions. We examined the distribution of coverage across contigs, linear scaffolds, and graph scaffolds. In the human metagenomic datasets analyzed here, the median coverage of contigs binned was 4.2× (Sharon), 19.5× (skin), and 23× (HMP). We found that graph scaffolds are not biased toward contigs that are more highly abundant (Supplementary Figure 5). In fact, graph scaffolds have the ability to bin variants that are usually lower coverage, simply because variants are linked to higher coverage neighbors.

We observed that binning results varied widely across samples. When samples had great strain diversity, like the mock community that contains over 100 different taxa, using graph scaffolds significantly improved the contiguity and quality of the bins. However, when samples were less diverse, like those in the Sharon dataset, all binning approaches produced similar results. The complexity and strain diversity of a sample have a significant impact on the effectiveness of binning, and on the improvement that can be obtained by leveraging variation-aware scaffolds.

Another advantage of working with variation-aware scaffolds in Binnacle is that the resulting bins contain a better representation of the genic content of the organisms from the sample. In our investigation of *C. acnes* in the skin microbiome, bins constructed from graph scaffolds contain a larger number of accessory genes than bins constructed from linear scaffolds or contigs. Furthermore, graph scaffold bins uniquely identified contigs in structural variants that were related to the CRISPR-Cas system, catabolic processes, transcriptional regulation, and cell wall biosynthesis; traditional binning approaches missed the association of these variants with this genome. We hope that this observation will further strengthen the case for the development and use of tools that explicitly model strain variation when analyzing metagenomic data sets.

It is important to note that while read-based binning approaches exist (Cleary et al., 2015; Kyrgyzov et al., 2020a), many metagenome binning methods, including Binnacle, can only work with assembled sequences from the sample. It has been shown that assembled sequences improve taxonomic classification (Tran and Phan, 2020). Generally, reads from rare species and low-coverage regions do not assemble well. Thus, binning methods may not be effective for low abundance species. Another important but often overlooked point is the variable resolution of bins obtained. Even though one would like to obtain all bins as species-level metagenome assembled genomes, this goal is rarely achieved in practice. First, it is important to note that the concept of a bacterial species is not well defined. Second, the level of sequence divergence between closely related organisms varies widely across the bacterial taxonomy and even across the length of genomes. This may explain the somewhat surprising observation that Binnacle maintains low bin contamination even when using graph scaffolds that include sequence variation. CheckM relies on the number of multicopy marker genes to compute contamination, and these genes are more likely to be conserved among the strains forming the pangenome represented by Binnacle bins. In mock communities, we were able to compute contamination more precisely by mapping contigs to the relevant reference genome sequences. Even in this setting, the use of graph scaffolds did not result in higher contamination levels. As we have noted earlier, the paired end information we used accurately linked together contigs from the same organism, i.e., the underlying scaffold information itself has a low level of contamination. We hypothesize that the longer context provided by scaffolds allows binning algorithms to more accurately detect relationships between sequences derived from a same organism, thereby leading to lower levels of contamination than when using contigs as a substrate for binning.

In its current implementation, Binnacle does not attempt to resolve the multiple strains/haplotypes represented in its bins. A number of algorithms developed for haplotype phasing (Low et al., 2020; Rhie et al., 2020), viral quasi-species estimation (Eriksson et al., 2008; Zagordi et al., 2010; Astrovskaya et al., 2011), and species estimation in metagenomics (Quince et al., 2017) can be applied here to estimate the number of species in a bin, and to split bins into multiple MAGs. We intend to pursue this line of research in future iterations of our tool.

We would also like to argue for the importance of effective visualization tools that can provide researchers with more information about the relative placement of contigs within a bin along a chromosome as well as variation information. Tools for visualizing assembly graphs, such as Bandage (Wick et al., 2015) and MetagenomeScope (Fedarko et al., 2017) are a first step in this direction, but these tools are still cumbersome to use in large data sets. Further opportunities for future research include new approaches for estimating depth of coverage, particularly when using data from multiple samples. While substantial progress has been made in the field of RNA-seq quantification [e.g., Salmon (Patro et al., 2017)], metagenomic approaches still rely on fairly simplistic assumptions.

We believe that Binnacle represents a first step toward the development of effective metagenomic analysis tools that can leverage all the information contained in one or more samples to reconstruct nearly complete genomic sequences, approaching the goal of automated reconstruction of MAGs.

## Data Availability Statement

All of the data used in this study was previously published and can be accessed from the NCBI SRA under BioProjects PRJNA376566 and PRJNA46333, and from http://gigadb.org/dataset/100719 and https://portal.hmpdacc.org/.

## Author Contributions

HM, NS, JM, and MP conceived the research project. HM and NS designed and implemented the algorithm, with the help of JM and MP. HM, NS, and JM analyzed the data. HM, NS, JM, and MP wrote the manuscript. All authors read and approved the final manuscript.

## Conflict of Interest

The authors declare that the research was conducted in the absence of any commercial or financial relationships that could be construed as a potential conflict of interest.

## Footnotes

1 https://ggkbase.berkeley.edu/carrol/organisms
