# Supplementary material for: Binnacle: Using Scaffolds to Improve the Contiguity and Quality of Metagenomic Bins
Source: Front Microbiol. 2021 Feb 24;12:638561. doi: 10.3389/fmicb.2021.638561 (PMC7945042; doi:10.3389/fmicb.2021.638561)
Supplement: Supplementary file 1 [file Data_Sheet_1.pdf]

Supplementary Materials

Additional results

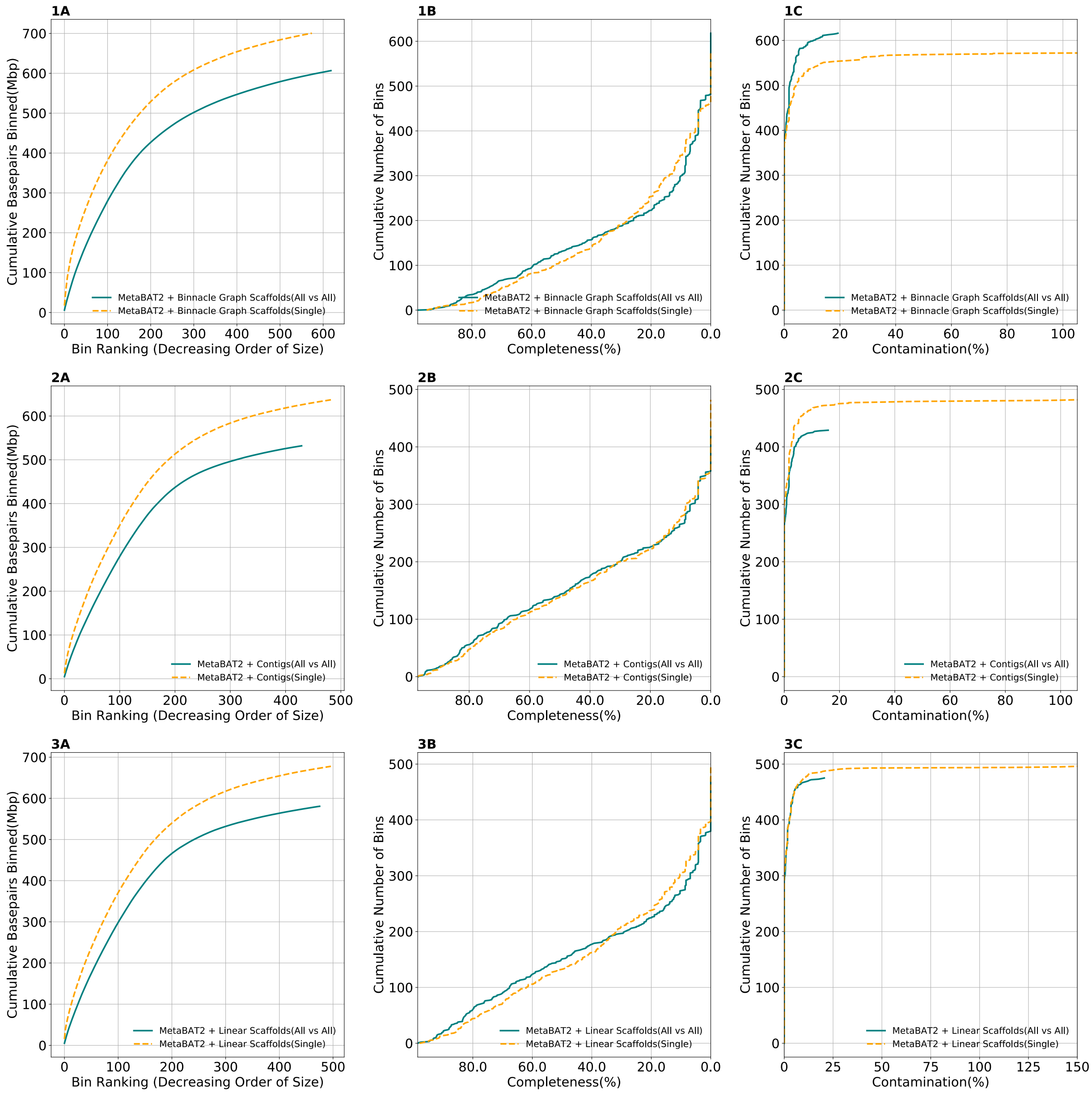

Figure S1. Binning using coverage information from all samples produces fewer high contamination bins for the HMP dataset.

Comparing bins generated by MetaBAT2 with graph scaffolds (1), contigs (2), and linear scaffolds (3) for the HMP dataset. A) Cumulative base pairs binned when using coverage from the single sample (yellow dotted line), and when using coverage information from all samples (blue solid line). Bins are ordered in decreasing order of their size. The upper curve corresponds to higher contiguity for the same number of bins. B) Bins are ordered in decreasing order of their

completeness value from CheckM evaluation. The upper curve indicates that more bins are contained at the same or higher level of completeness. C) Bins are ordered in the increasing order of their contamination value from CheckM evaluation. The higher curve indicates that more bins are contained at the same or lower level of contamination.

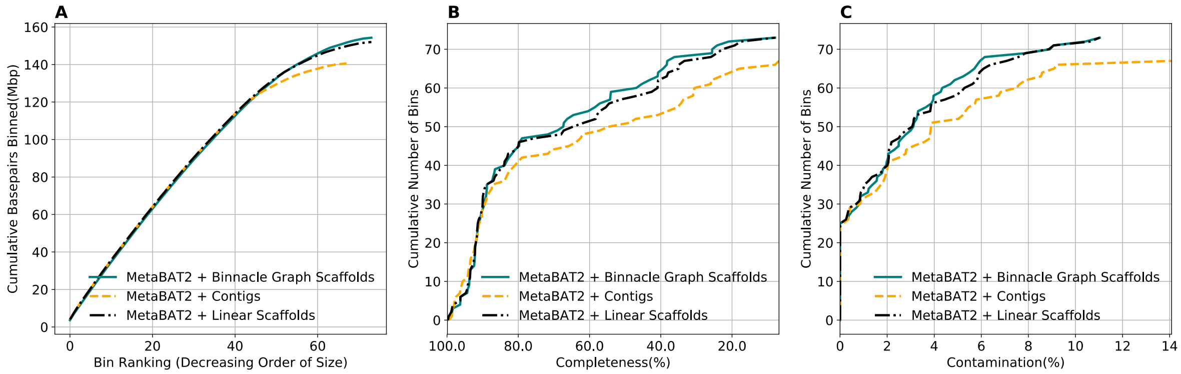

Figure S2. **Binning with graph scaffolds improves contiguity, completeness, and contamination in genome bins from the infant gut dataset.** Comparing bins generated by MetaBAT 2 using contigs, linear scaffolds, and graph scaffolds for the infant gut dataset. A) Cumulative base pairs binned with contigs, linear scaffolds, and graph scaffolds. Bins are ordered in decreasing order of their size. The upper curve corresponds to higher contiguity for the same number of bins. B) Completeness is defined as the percentage of the assigned genome represented in the bin. Bins are ordered in decreasing order of their completeness value. The upper curve indicates that more bins are contained in graph scaffolds at the same or higher level of completeness. C) Contamination of a bin is defined as the percentage of base pairs that did not align to the assigned genome. Bins are ordered in the increasing order of their contamination value. The higher curve indicates that more bins are contained in graph scaffolds at the same or lower level of contamination.

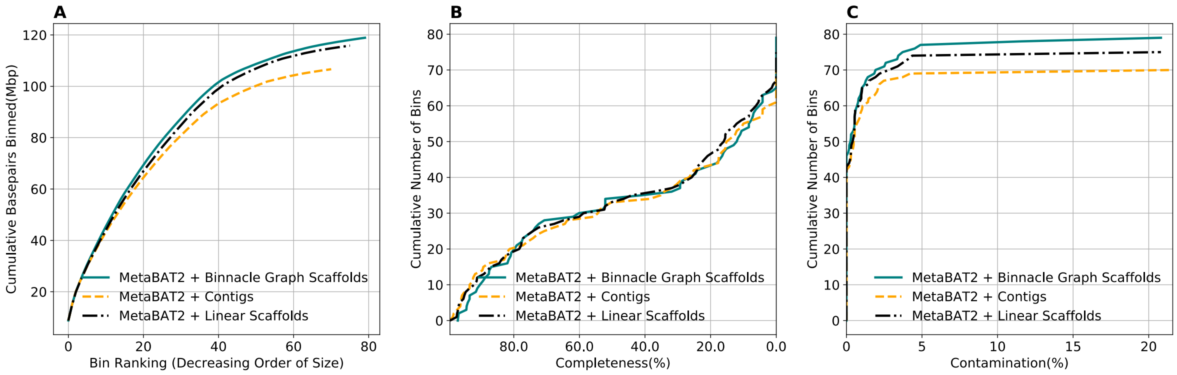

Figure S3. **Binning with graph scaffolds improves contiguity, completeness, and contamination in genome bins from the skin longitudinal study dataset.** Comparing bins generated by MetaBAT 2 using contigs, linear scaffolds, and graph scaffolds for the skin longitudinal study dataset. A) Cumulative base pairs binned with contigs, linear scaffolds, and graph scaffolds. Bins are ordered in decreasing order of their size. The upper curve corresponds to higher contiguity for the same number of bins. B) Bins are ordered in decreasing order of their completeness value from CheckM evaluation. The upper curve indicates that more bins are at the same or higher level of completeness. C) Bins are ordered in the increasing order of their contamination value from CheckM evaluation. The higher curve indicates that more bins are at the same or lower level of contamination.

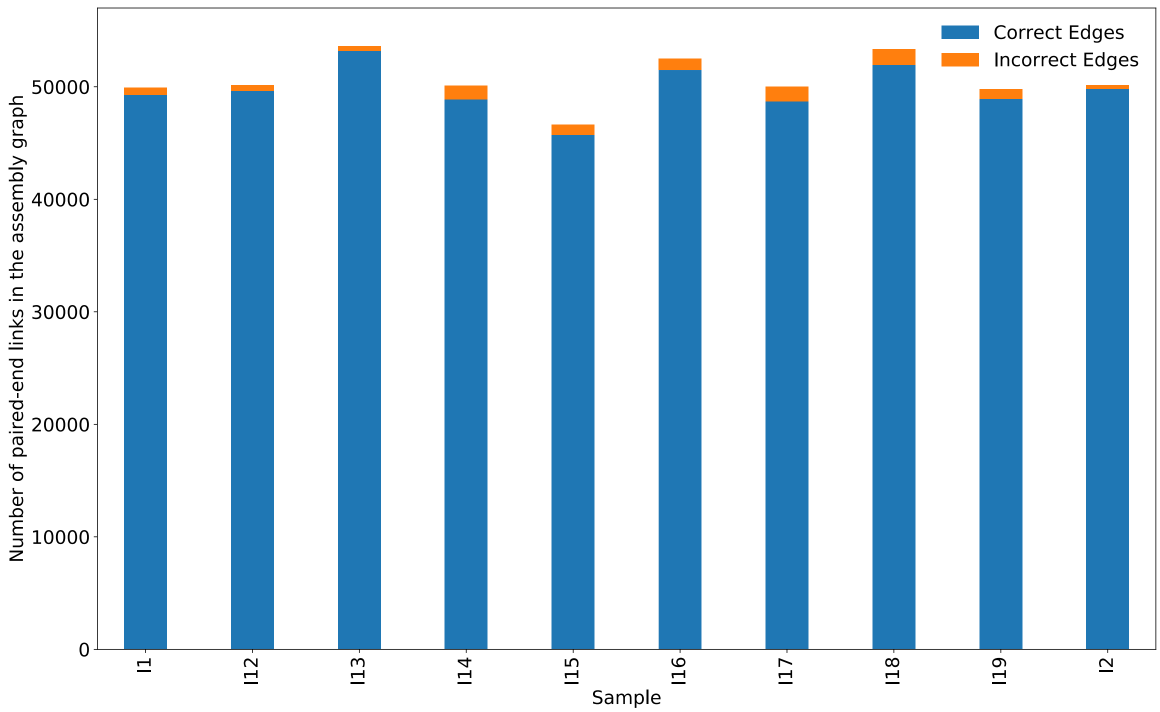

Figure S4. Error introduced by paired end linking information for the simulated dataset. The contigs obtained by assembling the reads of the simulated dataset were mapped to the reference genomes using minimap2. The species annotations in the contigs were then used to determine the fraction of correct edges generated by MetaCarvel.

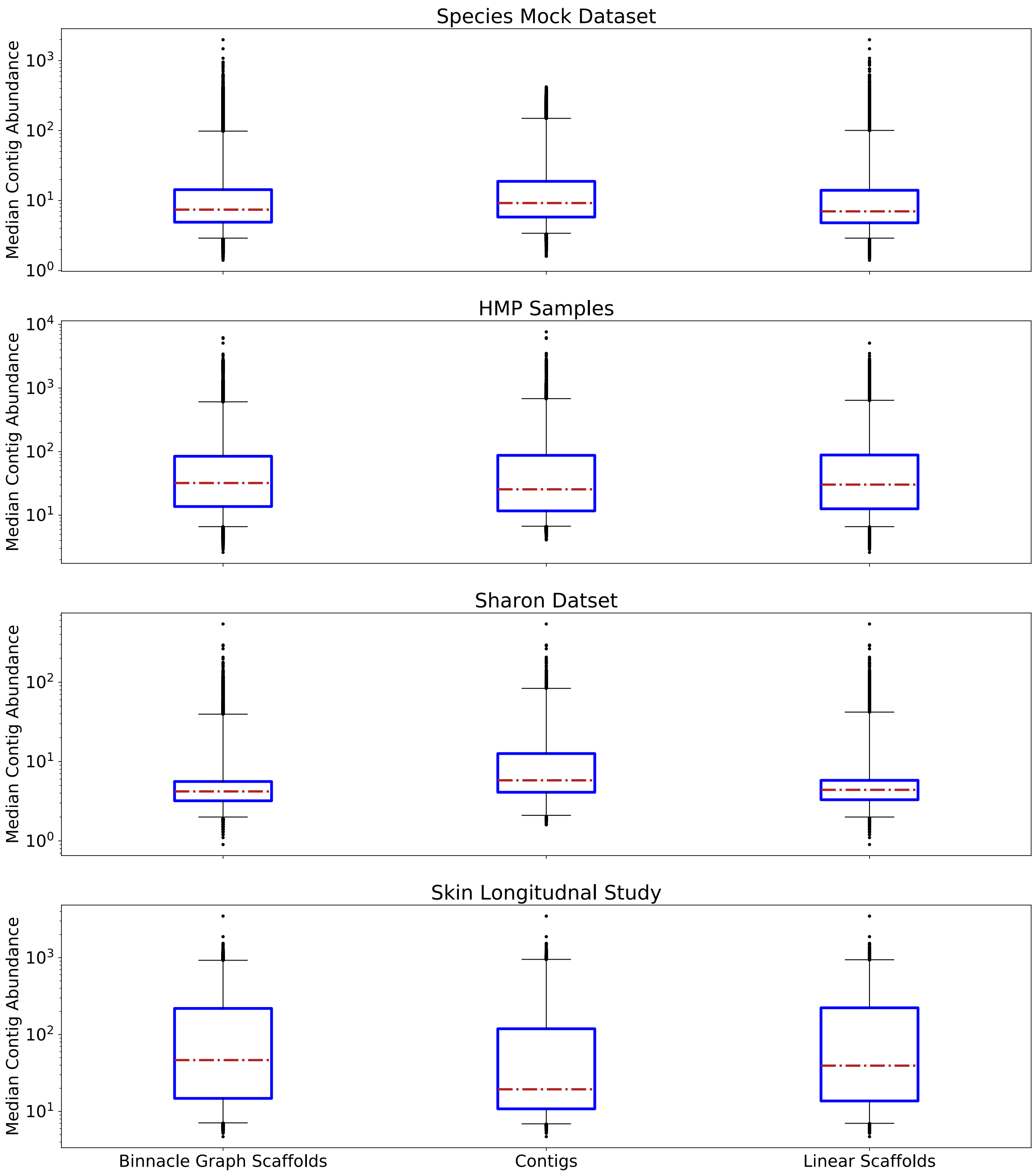

Figure S5. Boxplots of contig abundances binned by MetaBAT2 using graph scaffolds, linear scaffolds, and contigs for the different metagenomic datasets considered in this study.

Commands for methods used in the study

1. **Metagenome assembly**: For the HMP gut data, we downloaded assemblies provided by the HMP consortium. For the simulated, infant gut, and the skin longitudinal datasets, we assembled reads into contigs using MEGAHIT (version 1.1.2) with the following command
   - For infant dataset, skin longitudinal dataset
     - `megahit -1 [READS-1.fq.gz] -2 [READS-2.fq.gz] -o [OUTPUT_DIRECTORY] --out-prefix [SAMPLE_ID] --verbose`
   - For the mock community dataset
     - `megahit -1 [READS-1.fq.gz] -2 [READS-2.fq.gz] -o [OUTPUT_DIRECTORY] --out-prefix [SAMPLE_ID] --k-max [255] --verbose`

2. **Metagenome scaffolding:** For all datasets, we generated scaffolds using MetaCarvel
   - `python2 run.py -a [CONTIGS.fa] -m [MAP_READS_TO_CONTIGS.bam] -d $[OUTPUT_DIRECTORY] -k [TRUE] -r [TRUE]`
   - `python2 find_motifs.py -d [METACARVEL_OUTPUT_DIRECTORY]`

3. **Mapping reads to contigs**: We used bowtie 2 (version 2.3.0) to align reads to contigs, and use bedtools (version 2.26.0) to compute per-base coverage.
   - `bowtie2-build [CONTIGS.fa] [BOWTIE2_INDEX]`
   - `bowtie2 -x [BOWTIE2_INDEX] -U [READS_1.fq.gz,READS_2.fq.gz] --no-unal | samtools view -bS > [OUTPUT.bam]`
   - `samtools sort -n [OUTPUT.bam] -o [SORTED_OUTPUT.bam]`
   - `bamToBed -i [SORTED_OUTPUT.bam] | sort -k 1,1 >[OUTPUT.bed]`
   - `genomeCoverageBed -bga -split -i [OUTPUT.bed] -g [GENOME-LENGTH]>[COVERAGE.txt]`
   - `LC_ALL=C sort -k1,1 [COVERAGE.txt] > [COVERAGE_SORTED.txt]`

4. **Assigning genome of origin**: For the simulated and the infant gut dataset, we have the genome sequences of organisms present in the sample. For these sample, we align contigs to reference genomes using minimap2
   - `minimap2 --secondary=no -cx asm5 [REFERENCE.fa] [CONTIGS.fa] > [OUTPUT.paf]`

5. **Binnacle:** Following command was used to run Binnacle, and it outputs Binnacle graph scaffolds and their coverage estimates. Note that these scaffolds are different from the ones in the MetaCarvel output. `ORIENTED.gml` is available in the MetaCarvel output directory.

   - `python3 Estimate_Abundances.py -g [ORIENTED.gml] -a [COVERAGE_SORTED.txt] -c [CONTIGS.fa] -d [OUTPUT_DIRECTORY]`
6. **Binning the simulated data:** For the simulated data, we ran all three methods, MetaBAT2 (version 2.12.1), MaxBin 2.0 (version 2.2.5), and COCACOLA with a length filter of 2500 base pairs.
   - MetaBAT2 command
     - `Default length filter parameter: 2500`
     - `metabat2 -i [CONTIGS.fa] -o [OUTPUT_PREFIX] -l  -a [ABUNDANCES.txt] -v -d`

   - COCACOLA command
     - `Filter sequences by length before running COCACOLA`
     - `python cocacola.py --contig_file [CONTIGS.fasta] --abundance_profiles [ABUNDACNES.txt] --composition_profiles [TNF.txt] --edge_list [optional][EDGELIST.txt] --output [BINS.txt]`
     - `We specified edge list only while running COCACOLA with contigs.`

   - MaxBin 2.0 command
     - `run_MaxBin.pl -contig [CONTIGS.fa] -out [OUTPUT_DIRECTORY] -min_contig_length [2500] -thread [12] -abund_list [ABUNDANCES.txt]`

7. **CheckM evaluation**: For the HMP gut and the skin longitudinal data, we evaluate bin quality (completeness and contamination) using CheckM (version 1.0.11) program.
   - `checkm lineage_wf -t [12] -x fna [BINS_DIRECTORY] [OUTPUT_DIRECTORY]`
   - `checkm qa -t [12] -o [2] [CHECKM_OUTPUT_DIRECTORY/lineage.ms] [CHECKM_OUTPUT_DIRECTORY]/ > [CHECKM_STATS.txt]`

Supplementary Tables

Table S1. Samples analyzed from the three publicly available datasets.

Dataset	SampleID	BioSample	Description
Infant gut	SRR5406014	SAMN06444306	day of life 8
Infant gut	SRR5406013	SAMN06444307	day of life 10
Infant gut	SRR5406012	SAMN06444308	day of life 12
Infant gut	SRR5406011	SAMN06444309	day of life 14
Infant gut	SRR5406010	SAMN06444310	day of life 16
Infant gut	SRR5406009	SAMN06444311	day of life 18
Infant gut	SRR5406008	SAMN06444312	day of life 20
Infant gut	SRR5406007	SAMN06444313	day of life 21
Infant gut	SRR5406006	SAMN06444314	day of life 23
Infant gut	SRR5406005	SAMN06444315	day of life 27
Infant gut	SRR5406004	SAMN06444316	day of life 33
HMP gut	SRS012902	SAMN00034120	Stool
HMP gut	SRS013215	SAMN00034433	Stool
HMP gut	SRS016438	SAMN00037656	Stool
HMP gut	SRS019397	SAMN00040615	Stool
HMP gut	SRS019787	SAMN00041005	Stool
HMP gut	SRS020622	SAMN00041840	Stool
HMP gut	SRS023829	SAMN00045047	Stool
HMP gut	SRS024075	SAMN00045293	Stool
HMP gut	SRS024549	SAMN00045767	Stool
HMP gut	SRS053573	SAMN00073582	Stool
HMP gut	SRS054590	SAMN00074599	Stool
HMP gut	SRS057717	SAMN00077726	Stool
HMP gut	SRS064645	SAMN00083669	Stool
HMP gut	SRS077194	SAMN00087361	Stool
HMP gut	SRS098644	SAMN00095165	Stool
HMP gut	SRS104311	SAMN00099936	Stool
HMP gut	SRS104485	SAMN00100110	Stool
HMP gut	SRS140645	SAMN00139862	Stool
HMP gut	SRS143342	SAMN00142559	Stool
HMP gut	SRS143780	SAMN00142997	Stool
Skin longitudinal	MET0308	SAMN03025671	External auditory canal, visit 1
Skin longitudinal	MET0749	SAMN04360103	External auditory canal, visit 2

Skin longitudinal	MET0768	SAMN04360120	External auditory canal, visit 3
Skin longitudinal	MET0314	SAMN03025677	Occiput, visit 1
Skin longitudinal	MET0754	SAMN04360108	Occiput, visit 2
Skin longitudinal	MET0773	SAMN04360125	Occiput, visit 3
Skin longitudinal	MET0316	SAMN03025679	Plantar heel, visit 1
Skin longitudinal	MET0757	SAMN04360111	Plantar heel, visit 2
Skin longitudinal	MET0776	SAMN04360128	Plantar heel, visit 3
Skin longitudinal	MET0319	SAMN03025682	Toe web, visit 1
Skin longitudinal	MET0755	SAMN04360109	Toe web, visit 2
Skin longitudinal	MET0774	SAMN04360126	Toe web, visit 3

Table S2. ***Cutibacterium acnes*** **reference genomes used for pan genome analyses.**

**Accession**	**FTP download path**
GCA_000008345.1_ASM834v1	ftp://ftp.ncbi.nlm.nih.gov/genomes/all/GCA/000/008/345/GCA_000008345.1_ASM834v1/GCA_000008345.1_ASM834v1_genomic.fna.gz
GCA_004136195.1_ASM413619v1	ftp://ftp.ncbi.nlm.nih.gov/genomes/all/GCA/004/136/195/GCA_004136195.1_ASM413619v1/GCA_004136195.1_ASM413619v1_genomic.fna.gz
GCA_003030305.1_ASM303030v1	ftp://ftp.ncbi.nlm.nih.gov/genomes/all/GCA/003/030/305/GCA_003030305.1_ASM303030v1/GCA_003030305.1_ASM303030v1_genomic.fna.gz
GCA_008728435.1_ASM872843v1	ftp://ftp.ncbi.nlm.nih.gov/genomes/all/GCA/008/728/435/GCA_008728435.1_ASM872843v1/GCA_008728435.1_ASM872843v1_genomic.fna.gz
GCA_000231215.1_ASM23121v1	ftp://ftp.ncbi.nlm.nih.gov/genomes/all/GCA/000/231/215/GCA_000231215.1_ASM23121v1/GCA_000231215.1_ASM23121v1_genomic.fna.gz
GCA_004136215.1_ASM413621v1	ftp://ftp.ncbi.nlm.nih.gov/genomes/all/GCA/004/136/215/GCA_004136215.1_ASM413621v1/GCA_004136215.1_ASM413621v1_genomic.fna.gz
GCA_001469595.1_ASM146959v1	ftp://ftp.ncbi.nlm.nih.gov/genomes/all/GCA/001/469/595/GCA_001469595.1_ASM146959v1/GCA_001469595.1_ASM146959v1_genomic.fna.gz
GCA_003390995.1_ASM339099v1	ftp://ftp.ncbi.nlm.nih.gov/genomes/all/GCA/003/390/995/GCA_003390995.1_ASM339099v1/GCA_003390995.1_ASM339099v1_genomic.fna.gz
GCA_000217615.1_ASM21761v1	ftp://ftp.ncbi.nlm.nih.gov/genomes/all/GCA/000/217/615/GCA_000217615.1_ASM21761v1/GCA_000217615.1_ASM21761v1_genomic.fna.gz
GCA_001469615.1_ASM146961v1	ftp://ftp.ncbi.nlm.nih.gov/genomes/all/GCA/001/469/615/GCA_001469615.1_ASM146961v1/GCA_001469615.1_ASM146961v1_genomic.fna.gz
GCA_000240035.1_ASM24003v1	ftp://ftp.ncbi.nlm.nih.gov/genomes/all/GCA/000/240/035/GCA_000240035.1_ASM24003v1/GCA_000240035.1_ASM24003v1_genomic.fna.gz
GCA_001281065.1_ASM128106v1	ftp://ftp.ncbi.nlm.nih.gov/genomes/all/GCA/001/281/065/GCA_001281065.1_ASM128106v1/GCA_001281065.1_ASM128106v1_genomic.fna.gz
GCA_000302515.1_ASM30251v1	ftp://ftp.ncbi.nlm.nih.gov/genomes/all/GCA/000/302/515/GCA_000302515.1_ASM30251v1/GCA_000302515.1_ASM30251v1_genomic.fna.gz
GCA_011399455.1_ASM1139945v1	ftp://ftp.ncbi.nlm.nih.gov/genomes/all/GCA/011/399/455/GCA_011399455.1_ASM1139945v1/GCA_011399455.1_ASM1139945v1_genomic.fna.gz
GCA_000240055.1_ASM24005v1	ftp://ftp.ncbi.nlm.nih.gov/genomes/all/GCA/000/240/055/GCA_000240055.1_ASM24005v1/GCA_000240055.1_ASM24005v1_genomic.fna.gz
GCA_000025765.1_ASM2576v1	ftp://ftp.ncbi.nlm.nih.gov/genomes/all/GCA/000/025/765/GCA_000025765.1_ASM2576v1/GCA_000025765.1_ASM2576v1_genomic.fna.gz
GCA_003798465.1_ASM379846v1	ftp://ftp.ncbi.nlm.nih.gov/genomes/all/GCA/003/798/465/GCA_003798465.1_ASM379846v1/GCA_003798465.1_ASM379846v1_genomic.fna.gz
GCA_006739385.1_ASM673938v1	ftp://ftp.ncbi.nlm.nih.gov/genomes/all/GCA/006/739/385/GCA_006739385.1_ASM673938v1/GCA_006739385.1_ASM673938v1_genomic.fna.gz
GCA_000213155.1_ASM21315v1	ftp://ftp.ncbi.nlm.nih.gov/genomes/all/GCA/000/213/155/GCA_000213155.1_ASM21315v1/GCA_000213155.1_ASM21315v1_genomic.fna.gz

GCA_000709495.1_ASM70949v1	ftp://ftp.ncbi.nlm.nih.gov/genomes/all/GCA/000/709/495/GCA_000709495.1_ASM70949v1/GCA_000709495.1_ASM70949v1_genomic.fna.gz
GCA_003812785.1_ASM381278v1	ftp://ftp.ncbi.nlm.nih.gov/genomes/all/GCA/003/812/785/GCA_003812785.1_ASM381278v1/GCA_003812785.1_ASM381278v1_genomic.fna.gz
GCA_011399315.1_ASM1139931v1	ftp://ftp.ncbi.nlm.nih.gov/genomes/all/GCA/011/399/315/GCA_011399315.1_ASM1139931v1/GCA_011399315.1_ASM1139931v1_genomic.fna.gz
GCA_009177305.1_ASM917730v1	ftp://ftp.ncbi.nlm.nih.gov/genomes/all/GCA/009/177/305/GCA_009177305.1_ASM917730v1/GCA_009177305.1_ASM917730v1_genomic.fna.gz
GCA_000376705.1_ASM37670v1	ftp://ftp.ncbi.nlm.nih.gov/genomes/all/GCA/000/376/705/GCA_000376705.1_ASM37670v1/GCA_000376705.1_ASM37670v1_genomic.fna.gz
GCA_001469565.1_ASM146956v1	ftp://ftp.ncbi.nlm.nih.gov/genomes/all/GCA/001/469/565/GCA_001469565.1_ASM146956v1/GCA_001469565.1_ASM146956v1_genomic.fna.gz
GCA_001469655.1_ASM146965v1	ftp://ftp.ncbi.nlm.nih.gov/genomes/all/GCA/001/469/655/GCA_001469655.1_ASM146965v1/GCA_001469655.1_ASM146965v1_genomic.fna.gz
GCA_000240015.1_ASM24001v1	ftp://ftp.ncbi.nlm.nih.gov/genomes/all/GCA/000/240/015/GCA_000240015.1_ASM24001v1/GCA_000240015.1_ASM24001v1_genomic.fna.gz

Table S3. *Cutibacterium* contigs containing CRISPR/CAS Type I E genes and CRISPR arrays.

Sample	Contigs containing CAS Type 1E genes	Binning Methods Including CAS Type 1E genes	Contigs Containing Spacer Sequences	CRISPR Direct Repeat Consensus	CRISPR Spacer Sequences
MET0308	k119_4134	contig, graph scaffold	k119_9313 (4 evidence level)	GGCTCACCCCCGCATAGGCGGGGAATAC	>spacer1 TGCCGTCTCCGTCAAGGGGTACGTTCACGACGT >spacer2 GACATGGACCCCCAGGCCAATCTCGACATCGAC >spacer3 TACACCACTCATAGATACATTGAATCGGTGACA >spacer4 GGTGGTGGCGGCGTGTTGGGCTTGGGTGATTTG >spacer5 ACGAACTTGTTGTGGCCTTCTGAGATTGCTGCG >spacer6 AAGGATGCTGGTGCTTTGGGGGCTGCGTATGGG >spacer7 CGCTCAGCCAACATCGACTGCACCCCAGAAGAC
MET0749	k119_5525	contig, linear scaffold, graph scaffold	k119_7907 (4 evidence level)	GGCTCACCCCCGCATAGGCGGGGAATAC	>spacer1 TGCCGTCTCCGTCAAGGGGTACGTTCACGACGT >spacer2 TGCCGTCTCCGTCAAGGGGTACGTTCACGACGT >spacer3 GACATGGACCCCCAGGCCAATCTCGACATCGAC >spacer4 TACACCACTCATAGATACATTGAATCGGTGACA >spacer5 GGTGGTGGCGGCGTGTTGGGCTTGGGTGATTTG >spacer6 ACGAACTTGTTGTGGCCTTCT

					GAGATTGCTGCG >spacer7 AAGGATGCTGGTGCTTTGGG GGCTGCGTATGGG >spacer8 CGCTCAGCCAACATCGACTGC ACCCCAGAAGAC
			k119_7968 (1 evidence level) *not included in contig bins	AAGCTGA ACCAGCC TCGCATG GGAACA ACCG	>spacer1 ACAAACTTTTTCTCGACCTTG ACGGCGATT
MET07 68	k119_3355	contig, graph scaffold	k119_8570 (1 evidence level)	GTATTCC CCGCCTA TGCGGGG GTGAGCC	>spacer1 TGTCACCGATTCAATGTATCT ATGAGTGGTGTA >spacer2 GTCGATGTCGAGATTGGCCTG GGGGTCCATGTC >spacer3 ACGTCGTGAACGTACCCCTTG ACGGAGACGGCA
MET07 73	k119_9373	graph scaffold	k119_1847 (4 evidence level)	GGCTCAC CCCCGCA TAGGCGG GGAATAC	>spacer1 TGCCGTCTCCGTCAAGGGGTA CGTTCACGACGT >spacer2 GACATGGACCCCCAGGCCAA TCTCGACATCGAC >spacer3 CTCATGACTGACGACCCGACC TCACCCACCCAA >spacer4 TACACCACTCATAGATACATT GAATCGGTGACA >spacer5 GGTGGTGGCGGCGTGTTGGG CTTGGGTGATTTG
			k119_4580 (1 evidence level)	CTTGTGC CAATGAG TTCGAGG GGC	>spacer1 TACGTTTGGTTCGGTTTGCTG GAGCGGGCTGTCAAGA

Table S4. Running time and memory required for running Binnacle on the HMP dataset.

Sample	#Reads	Time(s)	Memory(GB)
SRS143780	140043911	5519.79	7.8
SRS104485	162343046	658.43	2.8
SRS104311	96184815	5805.27	9.8
SRS143342	78903046	1664.64	5.2
SRS140645	61183363	400.52	1.8
SRS098644	103061017	5514.41	10
SRS077194	110759083	2677.96	6.4
SRS064645	11097423	163.45	1.6
SRS057717	48910797	286.32	2.9
SRS054590	43128948	256.41	2.6
SRS053573	51106416	301.87	2.4
SRS024549	45098656	454.29	2.5
SRS024075	69350117	830.28	5.2
SRS023829	55521195	315	2.4
SRS020622	62291487	476.72	3.4
SRS019787	51351202	1072.6	4.8
SRS019397	54597908	1060.25	4.8
SRS016438	71667188	391.17	2.9
SRS013215	50440741	291.47	2.1
SRS012902	51103961	209.83	1.9
